# Supplementary material for: Pentoxifylline-induced protein expression change in RAW 264.7 cells as determined by immunoprecipitation-based high performance liquid chromatography
Source: PLoS One. 2022 Mar 25;17(3):e0261797. doi: 10.1371/journal.pone.0261797 (PMC8956197; doi:10.1371/journal.pone.0261797)
Supplement: S1 Fig — (PDF) [file pone.0261797.s001.pdf]

Supplementary data 1

Mathematical algorithm for IP-HPLC analysis

- IP-HPLC peak area (mAU*s) contains antigen peak ({Ag}), antibody peak ({Ab}), and antigen-antibody complex peak ({Ag-Ab}).

$A_{\text{(mAU*s)}} = \{Ag_1\} + \{Ab_1\} + \{Ag_2\text{-}Ab_2\}$

- UV spectrum shows relatively proportional value (α) between ({Ag} + {Ab}) and {Ag-Ab}; {Ag-Ab} = α({Ag} + {Ab})

$A_{\text{(mAU*s)}} = \{Ag_1\} + \{Ab_1\} + \alpha(\{Ag_2\} + \{Ab_2\})$

$A_{\text{(mAU*s)}} = (\{Ag_1\} + \alpha(\{Ag_2\}) +(\{Ab_1\} + \alpha\{Ab_2\})$

$\{Ag_1\} + \alpha(\{Ag_2\}_{\text{(mAU*s)}} = A - (\{Ab_1\} + \alpha\{Ab_2\})$

- When the antibody was monospecific or monoclonal to antigen, Ag1/Ag2 = Ab1/Ab2 = β.

$\{Ag_1\} + \frac{\alpha}{\beta}(\{Ag_2\}_{\text{(mAU*s)}} = A - (\{Ab_1\} + \alpha\{Ab_2\})$

$\{Ag_1\}\ (1 + \frac{\alpha}{\beta})_{\text{(mAU*s)}} = A - (\{Ab_1\} + \alpha\{Ab_2\})$

$\{Ag_1\}_{\text{(mAU*s)}} = \frac{\beta(A - (\{Ab1\} + \alpha\{Ab2\}))}{\alpha+\beta}$

And

$\{Ag_1\} + \alpha(\{Ag_2\}_{\text{(mAU*s)}} = A - (\{Ab_1\} + \alpha\{Ab_2\})$

$\beta(\{Ag_2\} + \alpha(\{Ag_2\}_{\text{(mAU*s)}} = A - (\{Ab_1\} + \alpha\{Ab_2\})$

$\beta(\{Ag_2\} + \alpha(\{Ag_2\}_{\text{(mAU*s)}} = A - (\{Ab_1\} + \alpha\{Ab_2\})$

$\{Ag_2\}(\alpha + \beta)_{\text{(mAU*s)}} = A - (\{Ab_1\} + \alpha\{Ab_2\})$

$\{Ag_2\}(\alpha + \beta)_{\text{(mAU*s)}} = \frac{A - (\{Ab1\} + \alpha\{Ab2\})}{\alpha + \beta}$

- And then, the objective antigen expression was $\{Ag_1\} + \{Ag_2\}$.

$$\{Ag_1\} + \{Ag_2\}_{\text{(mAU*s)}} = \frac{(1+\beta)A-2(\{Ab1\}+\alpha\{Ab1\})}{\alpha+\beta}$$

- $\{Ag_{1c}\} + \{Ag_{2c}\}$ (mAU*s) is an objective antigen expression of control group, while $\{Ag_{1e}\} + \{Ag_{2e}\}$ (mAU*s) is an objective antigen expression of experimental group.

- The ratio compared between experiment and control objective antigen expression is $(\{Ag_{1e}\} + \{Ag_{2e}\})/(\{Ag_{1c}\} + \{Ag_{2c}\})$.

$$\frac{\{\text{Ag1e}\}+\{\text{Ag2e}\}}{\{\text{Ag1c}\}+\{\text{Ag2c}\}} = \frac{Ae(1+\beta)-2(\{Ab1e\}+\alpha\{Ab2e\})}{Ac(1+\beta)-2(\{Ab1c\}+\alpha\{Ab2c\})}$$

$$= \frac{Ae-\frac{2(\{Ab1e\}+\alpha\{Ab2e\}}{1+\beta}}{Ac-\frac{2(\{Ab1c\}+\alpha\{Ab2c\}}{1+\beta}}$$

- $\frac{2(\{Ab1e\}+\alpha\{Ab2e\}}{1+\beta}$ and $\frac{2(\{Ab1c\}+\alpha\{Ab2c\}}{1+\beta}$ are replaceable with γA_{ave} (A_{ave} is average of A_c and A_e).

- Therefore,

$$\frac{\{\text{Ag1e}\}+\{\text{Ag2e}\}}{\{\text{Ag1c}\}+\{\text{Ag2c}\}} = \frac{Ae-\gamma Aave}{Ac-\gamma Aave}$$

- Because $(\{Ag_{1e}\} + \{Ag_{2e}\})$ and $(\{Ag_{1c}\} + \{Ag_{2c}\})$ are mathematically hypothetical value (mAU*s), their square root value may approximate the comparable expression level (mAU).

$$\sqrt{\frac{\{\text{Ag1e}\}+\{\text{Ag2e}\}}{\{\text{Ag1c}\}+\{\text{Ag2c}\}}} = \sqrt{\frac{Ae-\gamma Aave}{Ac-\gamma Aave}}$$

$$\frac{\textit{Experiment antigen expression level}}{\textit{Control antigen expression level}} = \sqrt{\frac{Ae-\gamma Aave}{Ac-\gamma Aave}} \text{ x 100 (\%)}$$

- γ can be determined by experimental IP-HPLC. If 15%-reduced amount of objective protein sample was applied to Protein A/G bead column compared to control group. The IP-HPLC results were as follow;

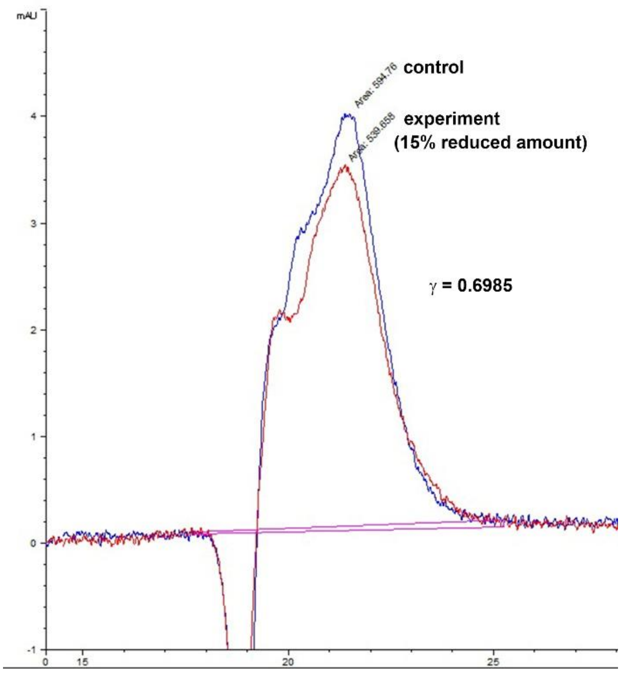

When the eluted proteins containing antibody were analyzed with 30 cm long column at low running speed (0.3 mL/min), the proteins were slightly separated but still appeared much overlapped in chromatography.

- And then,

$$\frac{Experiment\ antigen\ expression\ level}{Control\ antigen\ expression\ level} = \sqrt{\frac{Ae - \gamma Aave}{Ac - \gamma Aave}} \times 100 = 85\ (\%)$$

$$\sqrt{\frac{Ae - \gamma Aave}{Ac - \gamma Aave}} \times 100 = 85\ (\%)$$

$$\sqrt{\frac{539.658 - 567.209\gamma}{594.76 - 567.209\gamma}} \times 100 = 85\ (\%)$$

Therefore, constant γ can be calculated as 0.6985, and used to subtract other elements besides objective protein.

From this algorithm, the relative ratio (%) between objective protein level and control protein level can be obtained, albeit it is impossible to get the concentration of objective protein through IP-HPLC.
