# Supplementary material for: Pentoxifylline-induced protein expression change in RAW 264.7 cells as determined by immunoprecipitation-based high performance liquid chromatography
Source: PLoS One. 2022 Mar 25;17(3):e0261797. doi: 10.1371/journal.pone.0261797 (PMC8956197; doi:10.1371/journal.pone.0261797)

Supplementary data 2

Representative chromatography through IP-HPLC analysis

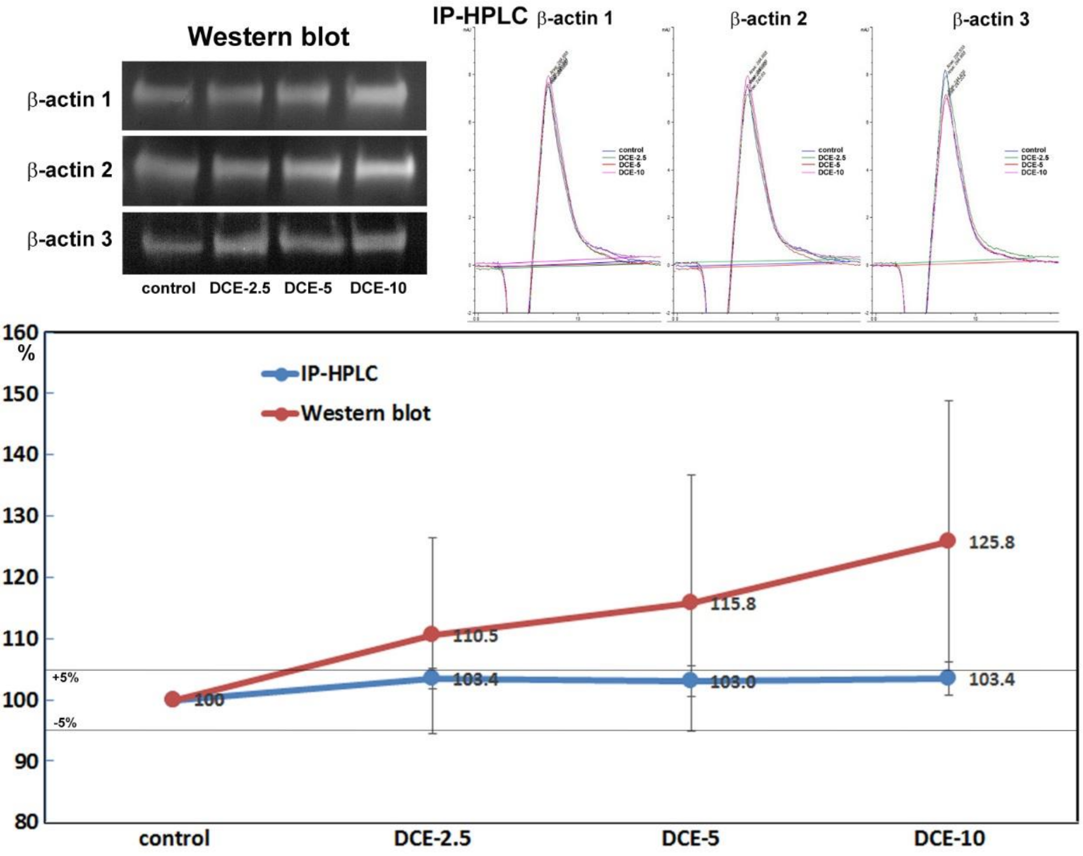

Supplementary Figure 2. β-Actin expression in DCE-treated RAW 264.7 cells was explored through western blot and IP-HPLC. Densitometry data of triplicated western blot (red line) showed big standard deviation (16.1 – 23.2 %), while triplicated IP-HPLC data (blue line) showed relatively small standard deviation (1.7 – 2.7%). Therefore, the latter was available to perform statistical analysis contrary to the former. These data were obtained from the previous study (Yoon, C.S., Kim, M.K., Kim, Y.S. & Lee, S.K. *In vitro* protein expression changes in RAW 264.7 cells and HUVECs treated with dialyzed coffee extract by immunoprecipitation high performance liquid chromatography. *Scientific reports* **8**, 13841 (2018))

Original digital images of Western blot

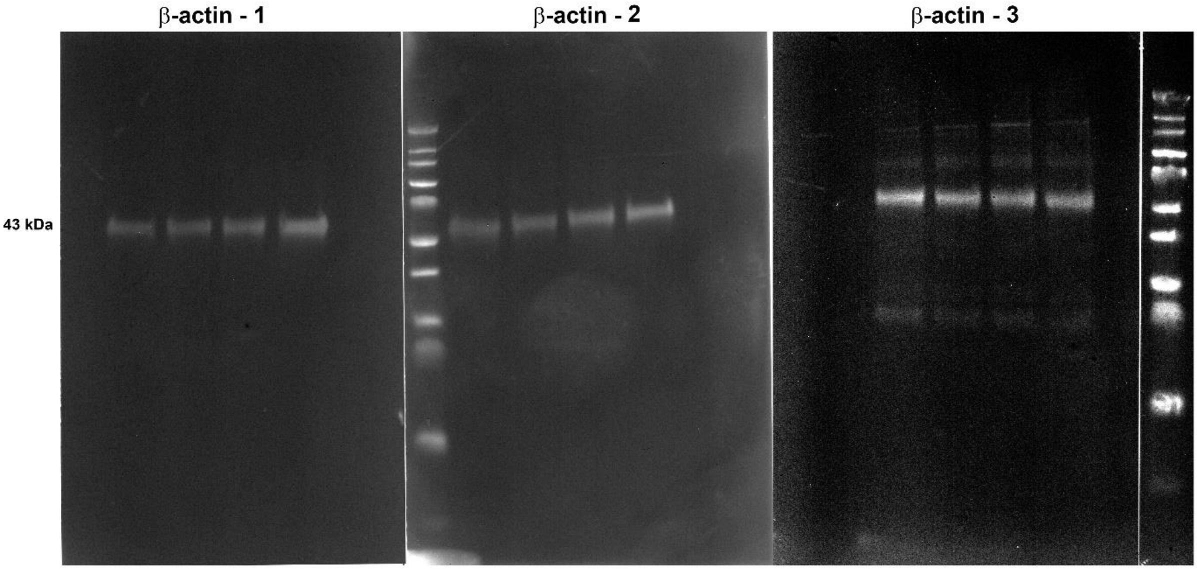

Supplement: S2 Fig — (PDF) [file pone.0261797.s002.pdf]
