# Supplementary material for: Pentoxifylline-induced protein expression change in RAW 264.7 cells as determined by immunoprecipitation-based high performance liquid chromatography
Source: PLoS One. 2022 Mar 25;17(3):e0261797. doi: 10.1371/journal.pone.0261797 (PMC8956197; doi:10.1371/journal.pone.0261797)
Supplement: Attachment — Submitted filename: Response to Reviewers-220302.docx [file pone.0261797.s005.docx]

**PLOS ONE Decision: Revision required [PONE-D-21-38689] - [EMID:ca32f9eaaa310396]**

- A rebuttal letter that responds to each point raised by the academic editor and reviewer(s). You should upload this letter as a separate file labeled 'Response to Reviewers'.

**Please, find a file named “Response to Reviewers-220302.docx”.**

- A marked-up copy of your manuscript that highlights changes made to the original version. You should upload this as a separate file labeled 'Revised Manuscript with Track Changes'.

**Please, find a file named “Revised Manuscript with Track Changes-PTX-220302.docx”.**

- An unmarked version of your revised paper without tracked changes. You should upload this as a separate file labeled 'Manuscript'.

**Please, find a file named “	“.**

If you would like to make changes to your financial disclosure, please include your updated statement in your cover letter. Guidelines for resubmitting your figure files are available below the reviewer comments at the end of this letter.

If applicable, we recommend that you deposit your laboratory protocols in protocols.io to enhance the reproducibility of your results. Protocols.io assigns your protocol its own identifier (DOI) so that it can be cited independently in the future. For instructions see: <https://journals.plos.org/plosone/s/submission-guidelines#loc-laboratory-protocols>. Additionally, PLOS ONE offers an option for publishing peer-reviewed Lab Protocol articles, which describe protocols hosted on protocols.io. Read more information on sharing protocols at <https://plos.org/protocols?utm_medium=editorial-email&utm_source=authorletters&utm_campaign=protocols>.

**[Answer]**

**Because the experimental protocols used in this experiment are generally simple and easy, it seems that no additional protocol is required.**

We look forward to receiving your revised manuscript.

Kind regards,

Michael Schubert
Academic Editor
PLOS ONE

Journal requirements:

When submitting your revision, we need you to address these additional requirements.

1. Please ensure that your manuscript meets PLOS ONE's style requirements, including those for file naming. The PLOS ONE style templates can be found at

<https://journals.plos.org/plosone/s/file?id=wjVg/PLOSOne_formatting_sample_main_body.pdf>  and

<https://journals.plos.org/plosone/s/file?id=ba62/PLOSOne_formatting_sample_title_authors_affiliations.pdf>

**[Answer]**

**The present manuscript has been readjusted to the fomatting sample file as indicated.**

2. PLOS ONE now requires that authors provide the original uncropped and unadjusted images underlying all blot or gel results reported in a submission’s figures or Supporting Information files. This policy and the journal’s other requirements for blot/gel reporting and figure preparation are described in detail at https://journals.plos.org/plosone/s/figures#loc-blot-and-gel-reporting-requirements and https://journals.plos.org/plosone/s/figures#loc-preparing-figures-from-image-files. When you submit your revised manuscript, please ensure that your figures adhere fully to these guidelines and provide the original underlying images for all blot or gel data reported in your submission. See the following link for instructions on providing the original image data: <https://journals.plos.org/plosone/s/figures#loc-original-images-for-blots-and-gels>.

In your cover letter, please note whether your blot/gel image data are in Supporting Information or posted at a public data repository, provide the repository URL if relevant, and provide specific details as to which raw blot/gel images, if any, are not available. Email us at plosone@plos.org if you have any questions.

**[Answer]**

**The raw western blot images are in Supporting Information. The western blot images of the present experiment were directly obtained from digital image analyzer of ChemiDoc XRS system (Bio-Rad Laboratories, Hercules, CA, USA), which were not followed by any image modification.**

3. We note that you have stated that you will provide repository information for your data at acceptance. Should your manuscript be accepted for publication, we will hold it until you provide the relevant accession numbers or DOIs necessary to access your data. If you wish to make changes to your Data Availability statement, please describe these changes in your cover letter and we will update your Data Availability statement to reflect the information you provide.

**[Answer]**

**Unfortunately, we have no repository accession number in use. Please, delete my previous statement of repository information.**

4. Thank you for stating the following financial disclosure:

“NO”

At this time, please address the following queries:

a)        Please clarify the sources of funding (financial or material support) for your study. List the grants or organizations that supported your study, including funding received from your institution.

b)        State what role the funders took in the study. If the funders had no role in your study, please state: “The funders had no role in study design, data collection and analysis, decision to publish, or preparation of the manuscript.”

c)        If any authors received a salary from any of your funders, please state which authors and which funders.

d)        If you did not receive any funding for this study, please state: “The authors received no specific funding for this work.”

Please include your amended statements within your cover letter; we will change the online submission form on your behalf.

5. Please check the correct figures 16 in pages 40 and 51.

**[Answer]**

**The authors received no specific funding for this work.**

**It was corrected, “Fig 19.” in page 52.**

6. Please review your reference list to ensure that it is complete and correct. If you have cited papers that have been retracted, please include the rationale for doing so in the manuscript text, or remove these references and replace them with relevant current references. Any changes to the reference list should be mentioned in the rebuttal letter that accompanies your revised manuscript. If you need to cite a retracted article, indicate the article’s retracted status in the References list and also include a citation and full reference for the retraction notice.

**[Answer]**

**No cited paper was retracted in this manuscript.**

[Note: HTML markup is below. Please do not edit.]

Reviewers' comments:

Reviewer's Responses to Questions

**Comments to the Author**

1. Is the manuscript technically sound, and do the data support the conclusions?

The manuscript must describe a technically sound piece of scientific research with data that supports the conclusions. Experiments must have been conducted rigorously, with appropriate controls, replication, and sample sizes. The conclusions must be drawn appropriately based on the data presented.

Reviewer #1: Yes

Reviewer #2: Yes

2. Has the statistical analysis been performed appropriately and rigorously?

Reviewer #1: N/A

Reviewer #2: Yes

3. Have the authors made all data underlying the findings in their manuscript fully available?

The [PLOS Data policy](http://www.plosone.org/static/policies.action#sharing) requires authors to make all data underlying the findings described in their manuscript fully available without restriction, with rare exception (please refer to the Data Availability Statement in the manuscript PDF file). The data should be provided as part of the manuscript or its supporting information, or deposited to a public repository. For example, in addition to summary statistics, the data points behind means, medians and variance measures should be available. If there are restrictions on publicly sharing data—e.g. participant privacy or use of data from a third party—those must be specified.

Reviewer #1: Yes

Reviewer #2: Yes

4. Is the manuscript presented in an intelligible fashion and written in standard English?

PLOS ONE does not copyedit accepted manuscripts, so the language in submitted articles must be clear, correct, and unambiguous. Any typographical or grammatical errors should be corrected at revision, so please note any specific errors here.

Reviewer #1: Yes

Reviewer #2: Yes

5. Review Comments to the Author

Please use the space provided to explain your answers to the questions above. You may also include additional comments for the author, including concerns about dual publication, research ethics, or publication ethics. (Please upload your review as an attachment if it exceeds 20,000 characters)

Reviewer #1: The "unbiased" large scale analysis of multiple signaling components at protein level adds new details to the mode of action, especially of the dose-dependent actions, of this old, but still used drug.

The weak point of the paper is that the results are based on a single mouse macrophage cell line. Since the agent is nowadays rarely used in mouse models, the considerable effort of the authors might be better spent using human models, better mimicking the clinical situation. Although analogous mature human macrophage cell lines are not available, the experiments might at least be supplemented some experiments with primary human macrophages or in vitro "matured" human monocyte cell lines.

The paper is still acceptable in its present form, however, the use of human primary cells or human cell lines would greatly increase its practical value.

**[Answer]**

**PTX is an old and well-known drug, but its effect of non-selective phosphodiesterase inhibitor may give an important roles in the treatment of different diseases. Recently, PTX is frequently recommended as a wound healing drug by inhibiting fibrosis and stimulating osteogenesis to prevent severe complications of osteoradionecrosis in head and neck cancer patients.**

# **Regarding the selection of human cell line, I am appreciate for your comment using primary human macrophages or in vitro "matured" human monocyte cell lines. It is really critical to use human therapeutic dose of PTX in human cell line, therefore, in the following experiment, we have a plan to perform by using human macrophages or Human Umbilical Vein Endothelial Cells (HUVECs). It is sorry to inform that we are unable to fulfill the reviewer’s comment at this time.**

Reviewer #2: The present work suggested that PTX has different biological effects on RWA 264.7 cells depending on the concentration of 10 μg/mL and 300 μg/mL PTX. The low dose 10 μg/mL PTX enhanced RAS/NFkB signaling, proliferation, differentiation, and inflammation, particularly, it stimulated neuromuscular and osteoblastic differentiation, innate immunity, and cell-mediated immunity, but attenuated ER stress, fibrosis, angiogenesis, and chronic inflammation, 44 while the high dose 300 μg/mL PTX was found to alleviate the 10 μg/mL PTX-induced biological effects, resulted in the suppression of RAS/NFkB signaling, proliferation, neuromuscular and osteoblastic differentiation, and inflammation. However, I have one comment to the authors as following:
- Could the authors explains what is the difference between this work and the recently published paper "Appl. Sci. 2021, 11, 8273. <https://doi.org/10.3390/app11178273>"?

**[Answer]**

**Dr. Kim, Soung Min, who is a corresponding author of the paper published in Appl. Sci. 2021, was cooperated with me for the PTX effect on RAW 264.7 cells. After the preliminary experiment, I found a great discrepancy between human therapeutic dose (10 μg/mL) of PTX and high dose (100 – 500 μg/mL) frequently used in many animal experiments. And then, Dr. Kim submitted the preliminary data to the Journal of Applied Science by inclining to the results reported in the literature in the absence of any discussion, therefore, I had retrieved my authorship from the paper.**

**The present manuscript is a revised and upgraded one through careful analysis using different methods. We hope this manuscript contribute a little advance in the elucidation of PTX pharmacological effect.**

6. PLOS authors have the option to publish the peer review history of their article ([what does this mean?](https://journals.plos.org/plosone/s/editorial-and-peer-review-process#loc-peer-review-history)). If published, this will include your full peer review and any attached files.


If you choose “no”, your identity will remain anonymous but your review may still be made public.


**Do you want your identity to be public for this peer review?** For information about this choice, including consent withdrawal, please see our [Privacy Policy](https://www.plos.org/privacy-policy).

Reviewer #1: No

Reviewer #2: No

[NOTE: If reviewer comments were submitted as an attachment file, they will be attached to this email and accessible via the submission site. Please log into your account, locate the manuscript record, and check for the action link "View Attachments". If this link does not appear, there are no attachment files.]

While revising your submission, please upload your figure files to the Preflight Analysis and Conversion Engine (PACE) digital diagnostic tool, <https://pacev2.apexcovantage.com/>. PACE helps ensure that figures meet PLOS requirements. To use PACE, you must first register as a user. Registration is free. Then, login and navigate to the UPLOAD tab, where you will find detailed instructions on how to use the tool. If you encounter any issues or have any questions when using PACE, please email PLOS at [figures@plos.org](mailto:figures@plos.org). Please note that Supporting Information files do not need this step.

**[Answer]**

**All figure files have been examined and converted by PACE digital diagnostic tool.**

**Besides the editor and reviewer’s comments, the present manuscript is added more IP-HPLC data using 6 antisera, including AXIN2, HMGB1, LGR4. Klotho, FOXO1, and FOXO3. Because the graph of protection proteins become much crowded and complicated, it is divided into two graphs of protection-related proteins and survival and aging-related proteins in Fig 13. And the diagram of global protein signaling pathway of PTX, Fig 19, is upgraded to demonstrate the panoramic scope of protein expression.**

**The additional minor correction of mistyped letter or sentences is done and traceable in “Revised Manuscript with Track Changes-PTX-220302.docx”.**

**Thank you very much for your kindness.**
